# Parental Mental Illness, Borderline Personality Disorder, and Parenting Behavior: The Moderating Role of Social Support

**DOI:** 10.1007/s11920-022-01367-8

**Published:** 2022-10-25

**Authors:** Fabian R. Seeger, Corinne Neukel, Katharina Williams, Marc Wenigmann, Leonie Fleck, Anna K. Georg, Felix Bermpohl, Svenja Taubner, Michael Kaess, Sabine C. Herpertz

**Affiliations:** 1 Department of General Psychiatry, Centre for Psychosocial Medicine, University of Heidelberg, Voßstraße 4, D – 69115 Heidelberg, Germany; 2 Department of Child and Adolescent Psychiatry, Centre for Psychosocial Medicine, University of Heidelberg, Heidelberg, Germany; 3 Department of Psychosocial Prevention, Centre for Psychosocial Medicine, University of Heidelberg, Heidelberg, Germany; 4 Department of Psychiatry and Psychotherapy, Charité Universitätsmedizin Berlin, Berlin, Germany; 5 University Hospital of Child and Adolescent Psychiatry and Psychotherapy, University of Bern, Bern, Switzerland

**Keywords:** Borderline personality disorder, Parenting behavior, Social support, Parental mental illness, Parenting interventions

## Abstract

**Purpose of Review:**

Parental mental disorders, particularly borderline personality disorder (BPD), impair parenting behavior. Consequently, the children exhibit an elevated risk for psychopathology across their lifespan. Social support for parents is thought to moderate the relationship between parental mental illness and parenting behavior. It may dampen negative effects and serve as starting point for preventive interventions. This paper provides a literature overview regarding the impact of social support on the sequelae of parental mental illness and BPD for parenting behavior.

**Recent Findings:**

Current literature highlights the increased burden of families with a mentally ill parent and associated changes in parenting behavior like increased hostility and affective dysregulation, especially in the context of parental BPD. Literature further demonstrates the powerful impact of social support in buffering such negative outcomes. The effect of social support seems to be moderated itself by further factors like socioeconomic status, gender, or characteristics of the social network.

**Summary:**

Social support facilitates positive parenting in mentally ill parents and may be particularly important in parents with BPD. However, social support is embedded within a framework of influencing factors, which need consideration when interpreting scientific results.

## Introduction

The quality of parenting behavior is frequently reported as the most influential environmental factor with regard to a child’s development [1, 2]. According to the widely referenced and scientifically supported process model of parenting by Belsky [3], parental personality and developmental history, social network, marital relations, work, and child characteristics jointly impact on parenting behavior. Taraban and Shaw [4•] recently updated the model by adding gender, cognitions and affect, stress response, genetics, emotion regulation, family structure, and culture as further factors and clustering them into the three domains, namely parent characteristics, child characteristics, and family and social environment. Overall, the model considers parenting as a buffered system, in which a risk factor might be compensated for by another positive or supportive influencing factor (i.e., resilience factor) across domains. Therefore, e.g., a challenging temperament of the child does not necessarily lead to impaired parenting quality if it is counterbalanced, e.g., by a supportive relationship between the parents [3, 4•]. On the other hand, a predominance of risk factors as well as the absence of sufficient resilience factors is thought to facilitate harsh, neglectful, or even abusive parenting [3, 4•].

In line with the process model, a highly influential risk factor with respect to negative parenting behavior is parental psychopathology, particularly BPD [4•, 5–7]. They relate to almost every influencing factor proposed within the model: e.g., parental personality [7], parental developmental history [8], social relationships [9], work ability, and socioeconomic status [9], as well as cognition, affect, stress response, and emotion regulation [10]. More specifically, important cognitive, emotional, or social prerequisites for positive parenting behavior such as attention, emotion regulation, or impulse control are often restricted in the presence of a personality disorder but also mental disorders in general [7, 11–13]. As a result, parents suffering from a mental disorder have been shown to exhibit greater difficulties in establishing authoritative parenting (the careful equilibrium of parental warmth and regulatory control; [14]). In contrast, parental psychopathology is thought to tip the balance towards permissive, rejecting-neglecting, or authoritarian parenting [14, 15]. Furthermore, our work group demonstrated in a previous study (UBICA-I) that mothers with a history of depression and severe early life maltreatment (ELM) show reduced maternal sensitivity, i.e., a less accurate and timed responsiveness to and perception of the child’s signals [16], when interacting with their child [17]. As maternal sensitivity appears to be especially diminished when mothers had experienced ELM and additionally suffered from a mental disorder in contrast to mothers who had experienced ELM but did not develop a mental disorder, parental psychopathology seems to be of special relevance when it comes to negatively altered parenting behavior [18]. With respect to personality disorders, maternal BPD has been associated with increased hostility towards the child, which further mediated the relationship between maternal BPD and behavioral problems in the child [19]. Additionally, parents with personality disorders like BPD report difficulties in establishing empathetic responsiveness towards the child, managing the child’s behavior, and being a role model for emotional regulation [20, 21].

These negative effects of parental mental illness on parenting behavior have been demonstrated across a variety of parental diagnoses like depression [4•], bipolar disorder [22], anxiety disorders [23], substance-use disorders [24], personality disorders in general [7], and BPD in particular [25–27]. Regardless of the exact underlying psychopathology, a reduced ability to correctly infer the child’s mental states such as emotions or psychological needs may additionally aggravate negative parenting behavior [6] and further heighten the risk for neglect, maltreatment, and abuse [28, 29].

For the child, the changes in parenting behavior might confer, e.g., to an increase in externalizing and internalizing problems [30–32], the development of insecure or disorganized attachment [33], depressive mood [32, 34], or dysfunctional social behavior [35], which in turn promote the risk for child psychopathology (for reviews, see [36, 37] and [38]). Changes in child functioning may in turn lead to parenting stress and altered parenting behavior due to transactional relations between child and parent variables [39]. In BPD, the formation of a healthy parent–child relationship as well as offspring emotional development have been shown to be impaired [33]. This results in a heightened risk for the children to develop a BPD themselves [33].

Taken together, parental mental illness, e.g., parental personality disorders such as BPD and the related changes in parenting behavior constitute an important factor in the intergenerational continuity of mental disorders besides genetic heritability of the individual diagnostic entities [36, 40]. Thirty-eight percent of physicians in German psychiatric hospitals report their patients to exhibit deficits in parenting behavior and roughly every second physician considers children’s mental health at risk due to those deficits [41]. As approximately three million German children live in families with at least one mentally ill parent, there is a high number of parents who may require additional support in dealing with their parental role [42].

This review aims to give an overview of the current literature on the sequelae of parental mental illness, specifically parental BPD, for parenting behavior as well as the moderating role of social support regarding this influence.

Literature search has been conducted between May 2021 and December 2021 via PubMed and Google Scholar. The following search terms were entered separately or in conjunction (respective manuscript section in parentheses): parental mental illness (1,2,4), parental mental disorder (1,2,4), personality disorder (1,2), borderline personality disorder (1,2), affective disorders (1,2), parenting (1,2,3,4), parenting behavior (1,2,3,4), social support (2,3,4), pandemic (3), SARS-CoV-2 (3), COVID-19 (3), preventive intervention (4), parenting intervention (4), parenting program (4). The literature was subsequently selected according to the year of publication (2000–2021).

## Social Support as a Moderator Between Parental Mental Illness, Parental BPD, and Parenting Behavior

In line with the process model [3], the accumulated stress of coping with a mental disorder and caring for a child simultaneously may impede symptom amelioration and positive parenting behavior [43, 44]. In turn, factors reducing parental stress have been observed to decrease severity of parental psychopathology as well as to promote positive parenting behavior [45•]. Social support may serve as such a factor of resilience [4•].

Moak and Agrawal [46] broadly defined social support as a psychosocial resource accessible in the context of the individual’s social network and interpersonal contacts. With respect to parenting, one may differentiate emotional, informational, and instrumental social support [47]. Emotional support mainly affects parenting behaviors indirectly via its effect on parental well-being, e.g., through the reduction of parental stress [45•, 48], the provision of a sense of social integration, or the aid in emotion regulation. Instead, informational and instrumental support also directly impacts on parenting behaviors, e.g., by promoting problem solving skills, providing advice or concrete aid in the accomplishment of everyday family requirements [49, 50]. Social support may therefore reduce the risk for child maltreatment via a decrease in parental stress and symptom severity as well as an increase in positive parenting behavior [45•, 51]. Furthermore, social support may reduce the negative effect of parental mental illness on child well-being [52] and promote service use in at-risk caregivers [53]. The results of Álvarez et al. [45•] further suggest a differential impact of social support obtained by professionals and institutions (formal support, [54]) and social support delivered by family members or friends (informal support): whereas informal support was more effective in changing child-rearing attitudes, formal support predicted a reduction in parental stress.

Research further highlights beneficial effects across different diagnoses, while the majority of studies focuses on parental depression: social support is associated with reduced parenting stress and lower levels of depression among parents [55, 56]. This effect has been demonstrated even before [57•] as well as shortly after child birth [58], thus serving as a protective factor against postpartum depression and bonding failure [57•]. Social support also seems to attenuate negative effects of parental depression on confidence in own parenting skills [59]. Within a longitudinal study spanning three generations, Abraham et al. [60••] found parental major depression to be a key factor accounting for the transmission of negative parenting behavior towards the next generation. This was mainly due to a parenting behavior characterized by reduced parental care to be transmitted to the children. However, individuals within the second generation did not carry on this behavior themselves if social support was present. Thus, social support aided in breaking the intergenerational cycle of negative parenting and parental depression.

Similar positive effects were described for patients with BPD, who often lack social support [21, 61]. Accordingly, social support has been demonstrated to moderate the influence of severity of several BPD symptoms like affective instability or identity problems on mothers’ emotional availability and thus a key feature of positive parenting [62].

However, there are also studies questioning that social support can be consistently regarded as having positive effects on parenting. For example, in BPD, the beneficial effect of social support on parental emotional availability was shown to mitigate with increasing BPD severity in parents [62]. Taraban et al. [63•] found the negative effect of maternal depression on parenting quality to be strongest in mothers reporting high levels of social support. Similarly, a recent study by Lee et al. [64] reported young mothers suffering from depression to be less able to benefit from social support. The authors of these studies speculated their findings might be due to depressed mothers with high social support delegating their parental responsibilities more often to their social network, which may prevent them from learning to keep high parenting quality in case of depressed mood [63•]. Alternatively, social support might provoke feelings of inadequacy due to prevalent cognitive biases in depressed mothers [64]. The findings were further supported by Taraban et al. [65•], who found the association between maternal depression and overreactive parenting to be unaffected by own satisfaction with social support. In contrast, the higher the partner’s satisfaction with social support was, the weaker the association between each parent’s depressive symptoms and overreactive parenting behavior. This finding further highlights a potential inability to take advantage of social support if parental depression is present. In substance-use disorders, increased parental social support has even been associated with more frequent physical abuse of the children by their parents [66].

However, social support does not only moderate the influence of parental mental illness on parenting behavior, but its effect is moderated itself by further influencing factors: in a study by Ceballo and McLoyd [67], the positive effects of social support on parenting behavior were attenuated with decreasing socioeconomic status in the environment of the participating mothers. Furthermore, there seem to be gender differences: Leinonen et al. [68] reported single fathers to be unable to benefit from emotional support by friends or relatives with respect to parenting behavior when facing economic strain. In contrast, single mothers were able to benefit from various sources of social support.

These findings emphasize the high complexity of influences with respect to parenting and social support as proposed by the process model of Belsky [3]. Additionally, the findings shed light on the methodological problem of a lacking consistent conceptualization of social support within science [69]. This leads to findings that seem to be contradictory at first, but emerge from different aspects or types of social support that have been studied (e.g., formal vs. informal support; support by relatives vs. support by friends). Therefore, there is a high need for a precise and generally acknowledged concept of social support in future studies [69].

**Fig. 1:**
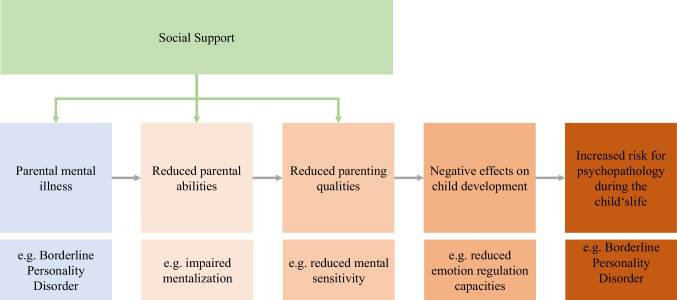
Exemplary model on the moderating role of social support on parenting behavior in mentally ill parents. The model illustrates a hypothesized pathway for the intergenerational continuity of mental disorders and potential starting points for the effects of social support

In summary, the majority of literature (for an overview see Table 1) points to substantial positive effects of social support on parenting behavior in the context of parental mental illness and across diagnostic categories. Unfortunately, we had to realize that research on social support particularly for parents with BPD is scarce, although parenting is severely affected in parents with BPD. Overall, social support may directly promote changes in parenting behavior or facilitate positive parenting via its effect on parental abilities and symptom severity (see Fig. 1). Therefore, it effectively hinders parental psychopathology from being forwarded to the child. However, the effects of social support may be moderated themselves by further (environmental) factors like socioeconomic status, characteristics of the social network, gender, or severity of symptomatology.

**Table 1:** Overview of reported studies on social support as a moderator of parenting behavior

Study	Type(s) of social support	Sample characteristics	Sample size	Results
Abraham et al. [60••]	Family & partner	Outpatients with MDD or HCs + their children & grandchildren	*N* = 498	Social support may break the continuity of negative MDD-associated parenting styles
Álvarez et al. [45•]	Formal & informal	Mainly at-risk families referred by municipal social services	*N* = 256	Informal support is associated with changes in child-rearing attitudes; formal support predicts reduction in parental stress
Armstrong et al. [50]	N/A	N/A	N/A	Social support as resilience factor with respect to parenting quality
Barnett et al. [47]	Perceived social-emotional support	Low-income mother–child dyads	*N* = 59	Parenting support is inversely related to parenting efficacy in mothers with stronger depressive symptoms
Ceballo and McLoyd [67]	Emotional & instrumental	Mother–child dyads from poor, high-crime environments	*N* = 262	Positive effects of social support on parenting behavior are attenuated in poor, high-crime environments
Cox et al. [59]	Affective	Adolescent mothers	*N* = 168	Depression is associated with decreased parenting confidence and decreased perceived social support
Crockenberg et al. [70]	N/A	N/A	N/A	Social support as buffer for stress and generator of active coping
Dunn et al. [61]	N/A	Parents with BPD Practitioners	*N* = 12 *N* = 21	High need for parenting-focused support in BPD patients
Freisthler et al. [66]	Social companionship	Telephone interviews on parenting and alcohol consumption in Californian parents	*N* = 3023	Social support may have negative effects: increased rates of physical abuse with increasing social companionship
Huang et al. [55]	Family/friends/significant other	African American or Latino/Hispanic adolescent mothers	*N* = 180	Lack of social support associated with negative mental health status and negative impacts on child development
Kang [53]	Affective & instrumental	Caregivers (incl. Grand- and stepparents)	*N* = 1000	Social support indirectly supports service use in at-risk caregivers
Leahy-Warren et al. [58]	Informational, instrumental, emotional, appraisal	First-time mothers shortly after childbirth	*N* = 410	Social support by family and friends substantially reduces maternal postpartum depressive symptomatology
Lee et al. [64]	Parent figure, partner	Young, low-income African American mothers	*N* = 192	Young mothers with depression have difficulties taking advantage of social support
Leinonen et al. [68]	Instrumental, emotional	Mothers/fathers	*N* = 1415	Mothers are able to benefit from various sources of social support, fathers only from instrumental support
Li et al. [71]	Confidant, affective, instrumental	Elementary school children	*N* = 405	Social support reduces the risk for child maltreatment
Liu et al. [48]	Family/friends/significant other	Wuhan medical staff and average citizens	*N* = 506	Depression and anxiety symptoms were inversely correlated with perceived social support during the pandemic
Maguire-Jack and Wang [51]	Friends/neighbors	Families	*N* = 1045	The higher neighborhood cohesion and social support, the lower parenting stress and levels of neglect
Milgrom et al. [56]	Attachment, social integration, opportunity for nurturance, reassurance of worth, reliable alliance, guidance relationships	Depressed women	*N* = 54	Social support in late pregnancy as protective factor against postpartum depression
Nunes et al. [52]	Confidant, affective, instrumental	Parents	*N* = 409	Parental affective support is predictive for child psychological adjustment
Ohara et al. [57•]	Quantity of supportive persons + satisfaction with support	Mothers shortly after childbirth	*N* = 494	Social support aids in preventing depression and bonding failure
Taraban et al. [63•]	Intimate relationships, friends, neighborhood/community	Mothers	*N* = 1096	Association between maternal depressive symptomatology and reduced parenting quality was strongest in the context of high social support
Taraban et al. [65•]	Intimate relationships, friends, neighborhood/community	Adoptive families	*N* = 519	Social support satisfaction of the partner reduces the strength of association between each parent’s depressive symptoms and overreactive parenting
Thompson [54]	Formal, informal	N/A	N/A	Informal support was more effective in changing child-rearing attitudes, formal support predicted a reduction in parental stress
Trupe [62]	Not specified	Mother–child dyads with and without maternal BPD	*N* = 70	The beneficial effect of social support on emotional availability was shown to mitigate with increasing BPD severity

## The SARS-CoV-2 Pandemic and Its Influences on Social Support and Parenting

Since SARS-CoV-2 has emerged in the end of 2019, the following pandemic and the related public health restrictions have fundamentally impacted on every individual’s social networks. Especially, regulations demanding social distancing have reduced possibilities for social support, thus withdrawing an important resource for families, especially those with at least one mentally ill parent. Studies point to long-lasting detrimental effects of the pandemic and related restrictions on mental health [72].

The loss of social support seems to be the crucial element in the exacerbated mental health burden of parents during the pandemic [73]. Correspondingly, there is already some evidence that the more social support was present, the less psychopathology increased during COVID-related lockdowns [74–76]. Social support may therefore constitute a starting point for interventions aiming at reducing the negative mental health impacts of the pandemic like a generally heightened level of psychological distress [76].

In line with the process model, this aggravation of parental symptomatology has facilitated negative parenting behaviors [77–79]. Accordingly, Sari et al. [80] reported heightened levels of harsh parenting during the pandemic and several studies have shown child mental health to decrease due in part to negatively altered parenting behaviors [81–84]. During COVID-19, parental depression has been determined to be a risk factor of negative parenting behaviors [85] which further aggravates in parents with a history of childhood maltreatment and a lack of compensatory resources such as social support [86].

As a result, Perks and Cluver [87] call for a “parenting vaccine” encompassing professional and scientifically evaluated parenting programs to buffer the negative effects of the pandemic on parental mental health, parenting behavior, and child well-being. Such programs might not only be beneficial for the individual who is participating, but may further aid in preventing negative and long-lasting socioeconomic and societal effects. The positive effects of parenting programs are further highlighted when considering the social support they implicate: if the interventions comprise formal support, e.g., via the teaching of parenting skills as well as informal support, e.g., by peer-to-peer exchange, their effects might be strengthened.

## Preventive Interventions Targeting Parenting Behavior

Parenting interventions enable the dissemination of positive parenting skills and thus allow for a direct impact on parenting behaviors of mentally ill parents [88••]. Besides, they may constitute an important source of social support for mentally ill parents. On the one hand, such interventions may provide formal social support via their association to specialized institutions and health care professionals. On the other hand, group programs may enable the exchange of experiences among parents and thus provide a valuable source of informal social support (see, e.g., [89]).

While the sole treatment of parental psychopathology has been shown to improve parenting behavior and child mental health, those interventions have only reached medium effect sizes (see, e.g., [90]). Directly targeting parenting behavior may be more effective due to its role as a mediator between parental and child mental health. Accordingly, numerous parenting programs aiming at improving parenting behavior have been developed in order to break the intergenerational continuity of mental disorders. Meta-analyses on such parenting programs have shown these interventions to effectively improve parenting behavior and child outcomes (see, e.g., [91–93]). Moreover, parenting programs also seem to prevent child maltreatment [94].

Recently, a meta-analysis by Everett et al. [88••] again highlighted that interventions targeting parenting behavior of mentally ill parents are successful in promoting positive parenting, but are especially effective in reducing child psychopathology. Furthermore, an improvement of parenting behavior diminished severity of parental psychopathology. The latter is thought to result from an improvement of parent–child interactions which account for reduced parental stress and thus facilitate symptom amelioration. The authors concluded that prevention programs not only need to address parenting behavior but also parental as well as child symptomatology to reach maximum efficacy [88••]. However, the exact pathways leading to the observed outcomes often remain unclear [95] and there is evidence that the efficacy of the programs varies significantly [96].

This variation might depend on the exact content of the parenting interventions: with respect to specific interventions, parental mentalization capabilities have been proposed to be a relevant prerequisite for positive parent–child interactions and positive parenting behavior [97]. Mentalization is defined as the ability to infer mental states within oneself and others [98]. Furthermore, maternal sensitivity has been demonstrated to be a promising starting point for parenting interventions [99].

Irrespective of the exact content of the intervention Marston et al. [100] found psychoeducation, peer-to-peer exchange of own experiences (an important source of informal social support), and skills for positive interactions within the family to be the three crucial elements of parenting interventions. Within this context, social support and peer-to-peer exchange may be also provided via online interventions [89], which gains special relevance during the pandemic. Furthermore, the use of video feedback may facilitate the observation of the child’s as well as one’s own behavior and thus provides a vital element to enhance preventive parenting programs [101]. Even though most preventive programs comprise psychoeducation elements, only few additionally provide opportunities for peer-to-peer exchange, specific interventions targeting parent–child interactions, and the use of video feedback.

A program that combines the three elements recommended by Marston et al. [100], incorporates video feedback, and thereby focuses on parental mentalization capabilities is the lighthouse parenting program [28] which is currently conducted and investigated in a study of our work group that aims to understand and break the intergenerational cycle of abuse (UBICA) in mentally ill parents [102]. The group program specifically focuses on social support via peer-to-peer exchange (informal social support) and further incorporates social counseling (formal social support). We test for superiority of this prevention program against pure psychoeducation and aim to identify potential mechanisms of change mediating the effects of the mentalization-based intervention on parenting behavior (for details see [102]).

## Conclusion

Parental mental illness and parental BPD influence parenting behavior in many ways with serious consequences for the offspring. The association between parental mental illness and problematic parenting behavior seems to be moderated by social support. Via its positive effect on parenting behavior, social support may also effectively aid in buffering or even preventing negative consequences for the children of mentally ill parents, and thus supports the discontinuation of mental illnesses and child maltreatment across generations. However, social support is embedded within a complex framework of influences on parenting behavior such as socioeconomic status, gender, or characteristics of the social network, that in turn moderate the effect of social support. Adding the lack of a generally acknowledged scientific conceptualization of social support, this leads to difficulties in comparing and interpreting research on social support in mentally ill parents. Future research should specify the type of social support that was investigated and consider potentially confounding factors, which may have moderated the influence of social support on their part.

In addition, specific research is needed to study the moderating role of social support within parents with BPD who seem to face aggravated problems in the context of parenting due to characteristics of their symptomatology. Especially, the increased hostility which is reported within the literature suggests a heightened need for (formal) support in those parents to improve emotion regulation and reduce negative effects on the children. This hypothesis crucially needs scientific evaluation.

Importantly, the majority of literature points to substantial positive effects of social support for families with a mentally ill parent. As the SARS-CoV-2 pandemic has isolated many families from social support, the need for structured and evidence-based parenting interventions has substantially increased. Optimally, such programs should comprise a combination of informal and formal support and should be broadly applied within standard clinical care of mentally ill parents to buffer long-term negative effects of the pandemic on parent and child mental health.
